# DIFFERENCES IN FRAILTY BETWEEN TRADITIONAL MEDICARE AND MEDICARE ADVANTAGE BENEFICIARIES

**DOI:** 10.1093/geroni/igad104.3279

**Published:** 2023-12-21

**Authors:** Sandra Shi, Brianne Olivieri-Mui, Ellen McCarthy, Dae Hyun Kim

**Affiliations:** Hebrew SeniorLife, Boston, Massachusetts, United States; Northeastern University, Boston, Massachusetts, United States; Hebrew SeniorLife, Boston, Massachusetts, United States; Harvard Medical School, Boston, Massachusetts, United States

## Abstract

Medicare Advantage (MA) plans cover at a minimum the same services as fee-for-service Medicare (FFS), but may target patient populations that differ in health and frailty. Leveraging 2011 and 2015 National Health and Aging Trends Study (NHATS) linked to Medicare claims, we classified participants as FFS or MA beneficiaries based on 12 months of Medicare enrollment. We calculated a deficit accumulation-based frailty index (FI) and phenotypic frailty from the survey assessment. All analyses accounted for the complex sampling design and weighted to reflect national estimates. In 2011, MA beneficiaries were more likely to be female (MA vs FFS: 59.1% vs 55.6%), Black (9.6% vs 7.4%) or other race (9.8% vs 5.2%), and to have an estimated income <$25,000 (44.5% vs 39.4%). Although the mean FI was similar between MA and FFS (MA vs FFS: 0.25 vs 0.25), MA beneficiaries were more likely to have mild frailty by FI (20.1% vs 18.6%) but less likely to have severe frailty by FI (10.0% vs 11.7%) or phenotypic frailty (13.5% vs 14.0%). MA beneficiaries had a higher prevalence of some comorbidities, such as hypertension (MA vs FFS: 64.9% vs 63.3%) and diabetes (24.4% vs 23.3%), but a lower mean number of ADL disability (0.32 vs 0.39). In 2015 the results were similar, although MA beneficiaries had a higher prevalence of pre-frailty (MA 46.0% vs 43.3%). In conclusion, MA beneficiaries seem to have a lower level of deficit-accumulation frailty and phenotypic frailty. This difference is driven by comorbidities, but not functional disability.

